# Variations in Sleep, Fatigue, and Difficulty with Concentration Among Emergency Medical Services Clinicians During Shifts of Different Durations

**DOI:** 10.3390/ijerph22040573

**Published:** 2025-04-06

**Authors:** Paul D. Patterson, Sarah E. Martin, Sean A. MacAllister, Matthew D. Weaver, Charity G. Patterson

**Affiliations:** 1 Department of Emergency Medicine, School of Medicine, University of Pittsburgh, Pittsburgh, PA 15213, USA; sam376@pitt.edu (S.E.M.); sam857@pitt.edu (S.A.M.); 2 Department of Community Health Services and Rehabilitation Science, School of Health and Rehabilitation Sciences, University of Pittsburgh, Pittsburgh, PA 15213, USA; 3 Department of Epidemiology, School of Public Health, University of Pittsburgh, Pittsburgh, PA 15213, USA; 4 Departments of Medicine and Neurology, Division of Sleep and Circadian Disorders, Brigham and Women’s Hospital, Boston, MA 02115, USA; mdweaver@bwh.harvard.edu; 5 Division of Sleep Medicine, Harvard Medical School, Boston, MA 02115, USA; 6 Department of Physical Therapy, School of Health and Rehabilitation Sciences, University of Pittsburgh, Pittsburgh, PA 15213, USA; cgp22@pitt.edu; 7 School of Health and Rehabilitation Sciences Data Center, University of Pittsburgh, Pittsburgh, PA 15213, USA

**Keywords:** shift work, fatigue, sleep

## Abstract

We sought to characterize momentary changes in fatigue, sleepiness, and difficulty with concentration during short and long duration shifts worked by emergency medical services (EMS) and fire personnel across the United States. In addition, we tested for differences in pre-shift and on-shift sleep stratified by shift duration. We examined real-time mobile-phone text message queries during scheduled shifts from the EMS Sleep Health Study, a nationwide, cluster-randomized trial (ClinicalTrials.gov Identifier: NCT04218279). Linear mixed effects models were used and Bonferroni *p*-values reported for multiple comparisons. In total, 388 EMS clinicians from 35 EMS/fire agencies documented 4573 shifts and responded to 64.6% of 161,888 text message queries. Most shifts (85.5%) were 12 or 24 h in duration. Mean sleep hours pre-shift was 6.2 (SD1.9) and mean sleep hours on shift was 3.4 (SD2.9) and varied by shift duration (*p* < 0.0001). The highest level of fatigue, sleepiness, and difficulty with concentration during any shift occurred during 24 h shifts and corresponded to the early morning hours at 03:00 or 04:00 a.m. The real-time assessments of sleep hours and fatigue in this study revealed deficits in sleep health for EMS and fire personnel and critical time points for intervention during shifts when the risk to safety is high.

## 1. Introduction

Fatigue and inadequate sleep are commonly reported and widespread problems among emergency medical services (EMS) clinicians and public safety personnel in the United States (U.S.) and other nations [1,2,3,4]. Our previous research in the U.S. showed that greater than half of EMS clinicians report poor sleep quality, three quarters report mental and physical fatigue, and half report inadequate recovery between scheduled shifts [3,5,6]. Fatigue among EMS clinicians increases the odds of injury, medical errors, and adverse events [3]. Previous research, including our own, largely lacked the granular assessments necessary to capture dynamic changes in fatigue that occur throughout shift work, especially for shifts of varying duration. A clearer understanding may be realized with the use of Ecological Momentary Assessment (EMA) techniques, which use repetitive assessments in rapid succession to detect subtle changes in outcomes like fatigue and sleepiness that may vary within a single shift [7,8]. Our prior research with EMA was limited to a restricted group of shift durations and a relatively small sample of EMS clinicians [9,10].

The primary objective of this study was to expand upon our previous research and provide new data on fatigue and fatigue-related measures like sleepiness assessed during shift work for a wide array of shift durations that represent the heterogeneity in shift scheduling approaches commonly used by EMS operations across the U.S. We report on pre-shift sleep and sleep obtained during shift work stratified by shift duration and include exploratory analyses for less common shift durations. Our overarching goal was to provide data that may be used by administrators of EMS and public safety operations and by researchers as base rate information regarding momentary changes in fatigue and sleep indicators for shifts of different durations, for comparison purposes, or for hypothesis generation for future research on EMS clinician fatigue and shift work.

## 2. Materials and Methods

### 2.1. Study Design and Population

We performed secondary analyses of data from the EMS Sleep Health Study, a nationwide, open-label, cluster-randomized wait-list control trial of U.S.-based EMS clinicians [2]. The source data come from participants reporting their sleep and feelings of fatigue using mobile-phone text message assessments. Text message assessments began February 2020; however, due to the onset of the COVID-19 pandemic, data collection was paused from March 2020 to June 2020. Data collection concluded on 31 July 2021. In total, 36 EMS agencies and 678 individual EMS clinicians were enrolled. Among the enrolled, 388 individuals affiliated with 35 EMS agencies had no exposure to intervention materials. In this analysis, we focused on these 388 individuals and the data they provided on a variety of real-world shifts over a 6-month study interval. The University of Pittsburgh Institutional Review Board approved this randomized clinical trial, and the trial protocol was registered with clinicaltrials.gov (NCT04218279) and the Office of Management and Budget (control number 20181-2127-003).

### 2.2. Data Collection

Participants received automated text message notifications and text message queries from the SleepTrackTXT platform [9,10,11,12]. The timing of the text message queries was based on the participants’ shift schedule, which was documented by the participant on a secure, password protected, online calendar. Like previous research [9,10,11,12], the text message protocol adhered to the principles of EMA [7,8] with the queries sent at the start, every 4 h during, and at the end of scheduled shifts. The platform sent text queries for 1-week (“on”) followed by 2 weeks without text queries (“off”), to avoid overly burdening participants. These queries were intended for data collection only and were not a component of the intervention.

### 2.3. Outcome Measures

Outcome measures reported by text message included: (1) hours of sleep achieved prior to shift start (within the past 12 h); (2) sleepiness; (3) fatigue; (4) difficulty with concentration; and (5) hours of sleep achieved during the scheduled shift (see the Supplemental File S1 for the text message prompts). Participants were instructed to report sleep in half hour increments (e.g., 6.5), and to rate sleepiness, fatigue, and difficulty with concentration “now”, for example: “Rate SLEEPINESS now (0–5) with 0 = Not at all and 5 = Very much”. Text message queries regarding sleepiness, fatigue, and difficulty with concentration were modeled after previous research [13], and have been used in multiple randomized trials of EMS clinicians [9,10,11,12].

### 2.4. Independent Measures of Interest

Shift duration was our primary independent variable of interest. We defined shift duration by first examining shift schedules documented by participants and grouping these shifts into 9 commonly used, practical categories: 8 h shift (≤9.49 h), 10 h shift (9.5 to 10.99 h), 12 h shift (11.0 to 13.99 h), 16 h shift (14 to 19.99 h), 24 h shift (20 to 29.99 h), 36 h shift (30 to 40.99 h), 48 h shift (41 to 54.99 h), 60 h shift (55 to 65.99 h), and 72 h shift (≥66 h). For the selected shift durations, we stratified the shifts by start time into AM (antemeridian 00:00–11:59) and PM (postmeridian 12:00–23:59). We also collected data at shift end on the number of patients seen during their shift (patient volume) and the number of hours slept (napped) during shift work. The text query regarding patient volume was presented to participants as: “Estimate the total number of patients you saw during your shift”. The text query regarding on-shift sleep was presented to participants as: “How many hours did you sleep DURING your shift (specify in half hours, ex. 2.5. No sleep = 0)”. As described in the statistical analysis section below, we examined the impact of patient volume and on-shift sleep on outcomes of interest.

### 2.5. Power Calculation and Sample Size Estimation

The original EMS Sleep Health Study was powered on the primary survey outcome measure of interest, the Pittsburgh Sleep Quality Index (PSQI) instrument, which was administered at baseline, 3 months, and 6 months [2]. The analyses reported in this paper were not guided by a formal power calculation to detect an a priori change or difference in text message assessments.

### 2.6. Statistical Analysis

We used graphs and descriptive statistics (e.g., frequencies and percentages) to describe the number of shifts documented by participants. We examined the mean hours of sleep before the scheduled shift, mean hours of sleep during the shift, and mean sleepiness, fatigue, and difficulty with concentration at the start, every 4 h during, and at the end of each shift. The median, interquartile range, and minimum and maximum values are also reported in the figures. We used linear mixed effects models with agency clusters and participants within clusters treated as random effects to test for differences in pre-shift and on-shift sleep by shift duration. Specific to the 8 h, 10 h, 12 h, and 24 h shifts, we tested for differences in sleepiness, fatigue, and difficulty with concentration at the start, during, and end of shifts by day shift (AM) versus night shift (PM). Bonferroni adjusted *p*-values are reported to account for multiple comparisons. Comparisons were omitted when cell sizes for selected time points and outcomes included <30 observations, given the risk of producing unstable estimates [14]. Linear mixed models did not include adjustments for demographic factors. We also used linear mixed models to examine the effect of patient volume during 24 h shifts on intra-shift sleep length, fatigue, sleepiness, and difficulty with concentration. Patient volume was based on the self-reported number of patient contacts via text message responses at the shift end. Patient volume was categorized into quartiles for the linear mixed model. Given the high prevalence of missing data for patient volume on non-24 h shifts, analyses accounting for patient volume were restricted to 24 h shifts. Additional analyses included linear mixed models to examine the impact of on-shift sleep as a predictor for end-of-shift assessments. These analyses were isolated to 12 or 24 h shifts and were stratified by shift start time (AM/PM). Exploratory analyses of shift durations that represented less than 5% of all shifts in this dataset (i.e., 16 h, 36 h, 48 h, and 72 h shifts) are reported in the Supplemental Files. All statistical analyses were performed with SAS version 9.4 (SAS Institute Inc., Cary, NC, USA).

## 3. Results

### 3.1. Baseline Data

**Figure 1:**
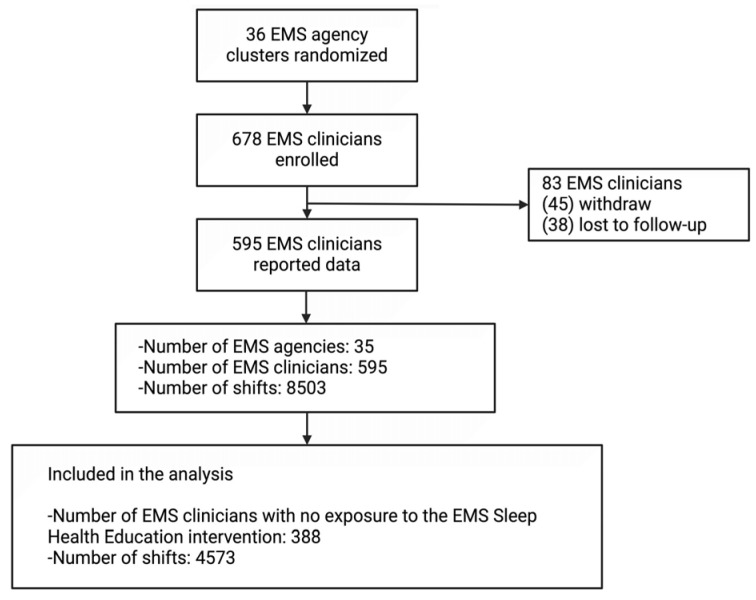
Flow diagram. EMS = emergency medical services. Figure 1 shows the flow of data collected from the EMS Sleep Health Study and what data were used in the secondary analyses presented in this manuscript.

Data were available for 388 EMS clinicians (Figure 1). Participants’ mean age was 38.2 years (SD 9.8) and mean years of experience was 13.7 years (SD 9.3). Most participants were male (69.1%), paramedic certified (61.1%), worked ground-based EMS (86.6%), employed full-time (94.6%), were mostly White race (90.2%), and not Hispanic or Latino (89.7%). Nearly one-quarter (23.2%) reported working multiple jobs.

### 3.2. Shifts Analyzed

A total of 4573 shifts were documented and the mean number of shifts per clinician was 9.3 (SD 7.1, min 1, max 45). Table 1 shows the distribution of shifts by duration, among which 85.5% were 12 or 24 h in duration. Most shifts (78.4%) had AM start times. Two-thirds of all 12 h shifts started in the AM, with the most common start time at 06:00 and at 18:00 for PM shifts. The most common start time for 24 h shifts was 08:00.

### 3.3. Pre-Shift Hours of Sleep

**Figure 2:**
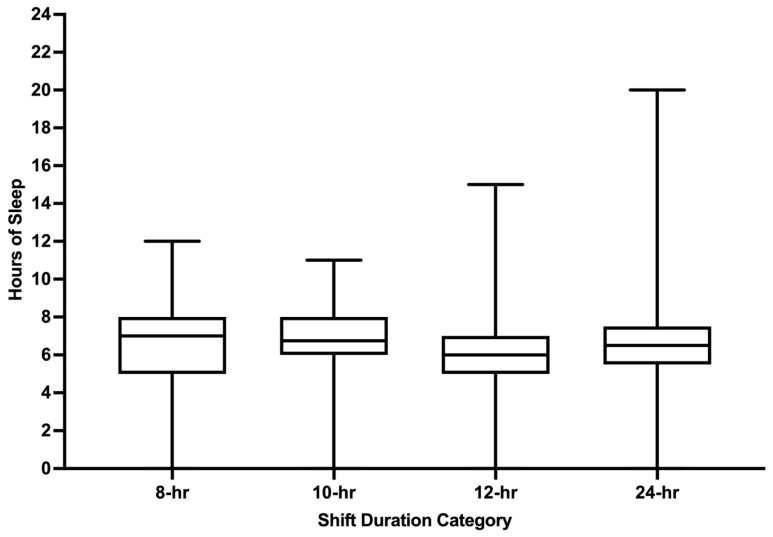
Pre-shift sleep hours by shift duration. hr = hour. Whiskers represent minimum and maximum. Boxes represent the interquartile range. Median is represented by the bar in the middle of the box.

**Figure 3:**
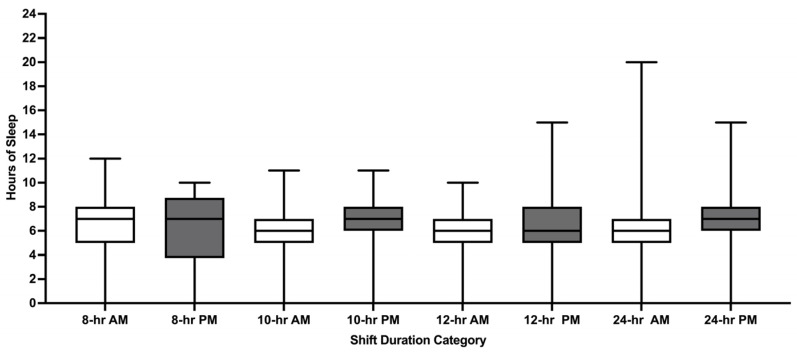
Pre-shift sleep hours stratified by shift duration and by AM/PM shift start time. hr = hour. AM = antemeridian (00:00–11:59). PM = postmeridian (12:00–23:59). Whiskers represent minimum and maximum. Boxes represent the interquartile range. Median is represented by the bar in the middle of the box.

Pre-shift hours of sleep were reported for 65.7% of 4401 shifts. Mean pre-shift sleep was 6.2 h (SD1.9) and varied by shift duration with greater sleep reported before 24 h shifts versus 8 h, 10 h, and 12 h shifts (all *p* values < 0.05; Figure 2). Pre-shift sleep of ≤6 h was reported for 51.3% of shifts. Mean pre-shift sleep hours also varied by AM/PM start times for 24 h shifts (*p* = 0.0392; Figure 3). Except for 8 h shifts, participants reported greater pre-shift sleep for shifts with PM start times versus AM start times (all *p* values < 0.05).

### 3.4. Hours of Sleep Obtained During Shift Work

**Figure 4:**
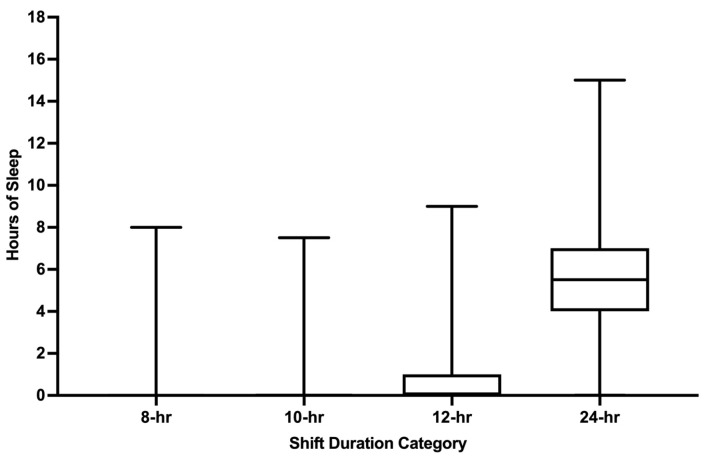
End of shift sleep hours stratified by shift duration. hr = hour. Whiskers represent minimum and maximum. Boxes represent the interquartile range. Median is represented by the bar in the middle of the box.

**Figure 5:**
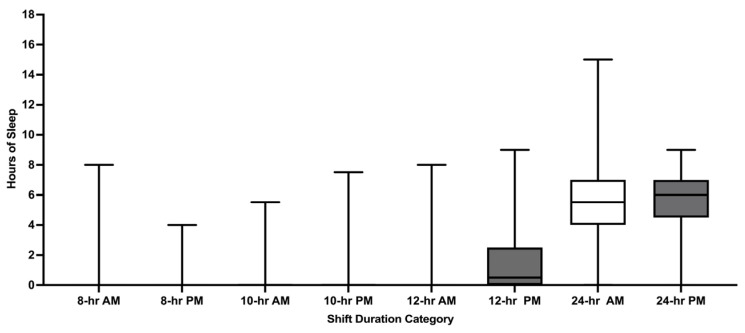
End of shift sleep hours stratified by shift duration and by AM/PM shift start time. hr = hour. AM = antemeridian (00:00–11:59). PM = postmeridian (12:00–23:59). Whiskers represent minimum and maximum. Boxes represent the interquartile range. Median is represented by the bar in the middle of the box.

Mean hours of sleep during shift work across all shifts examined was 3.4 h (SD 2.9) and increased with increasing shift duration; the greatest amount of sleep was documented during 24 h shifts (all *p* values < 0.01; Figure 4). Sleep during 10 h shifts (0.9 h) was not significantly greater than sleep during 8 h shifts (0.6 h; *p* = 1.00) nor was sleep during 10 h shifts (0.9 h) significantly greater than sleep during 12 h shifts (1.3 h; *p* = 0.1184). For the shifts where participants reported ≤6 h of pre-shift sleep, the mean on-shift sleep hours was 3.2 (SD 2.7). Nearly one-third of participants (30.2%) who reported ≤6 h of pre-shift sleep also reported no sleep during their shift. Only 12 h shifts had greater on-shift sleep during PM scheduled shifts compared to AM scheduled shifts (Figure 5; *p* < 0.0001). No AM/PM differences in on-shift sleep length were detected for 8 h, 10 h, or 24 h shifts (all *p* values > 0.05). Among participants who reported the number of patient encounters during 24 h shifts, an increasing number of patient encounters was strongly associated with less sleep during shift work (*p* < 0.0001; Supplemental Figure S6).

### 3.5. Fatigue During Shifts

Mean fatigue reported at the start of shifts for all 8 h, 10 h, 12 h, and 24 h shifts was low at 1.6 (SD 1.5), and there were no differences by shift duration (all *p* values > 0.05; Supplemental Figure S7). A pattern of increased fatigue with increasing hours on shift was detected for most shift durations, except for 24 h shifts, which showed a sharp increase in fatigue at 16 h into the shift, followed by a sharp decrease at shift end (Supplemental Figure S7). Fatigue at shift end was higher than shift start for all shift durations (all *p* values < 0.05); however, the increase (the delta) from start to end did not differ by shift duration (*p* = 0.1096). Supplemental Figure S8 shows differences in fatigue at the start, during, and at the end of 8 h, 10 h, 12 h, and 24 h shifts based on when the shift began, in the AM or PM. The AM/PM differences in fatigue during a 10 h shift occurred at the 8th hour of the shift. The AM/PM differences in fatigue during a 12 h shift occurred at the 8th hour and 12th hour time points (all *p* values < 0.05). No AM/PM differences in fatigue were detected for 8 h, or 24 h shifts (all *p* values > 0.05). Among participants who reported the number of patient encounters during 24 h shifts, the greater the patient encounters, the greater fatigue at all assessment time points during the shift (*p* < 0.0001; Supplemental Figure S9). On-shift sleep during 12 h and 24 h shifts was associated with lower fatigue at the end of the shift (*p* < 0.0001; Supplemental Table S1). The effect of on-shift sleep on fatigue was 3.6 times larger for 24 h shifts than 12 h shifts (*p* < 0.0001) and did not differ by AM/PM shift start time.

### 3.6. Sleepiness During Shifts

Participant-reported sleepiness and difficulty with concentration during shift work followed patterns like that observed for fatigue. Like fatigue, mean sleepiness reported at the start of a shift for all 8 h, 10 h, 12 h, and 24 h shifts was low at 1.7 (SD 1.5); and there were no between-shift differences detected (all *p* values > 0.05; Supplemental Figure S7). However, a pattern of increased sleepiness with increasing hours on shift was detected for most shift durations, except for 24 h shifts, which showed a sharp increase in sleepiness at 16 h into the shift, followed by a sharp decrease at shift end (Supplemental Figure S7). Sleepiness at shift end was higher than at shift start for all shift durations except 8 h shifts (all non-8 h shift *p* values < 0.05); however, like the pattern observed with fatigue, the increase (the delta) from start to end did not differ by shift duration (*p* = 0.0940). Supplemental Figure S10 shows the differences in sleepiness during and at the end of the 8 h, 10 h, 12 h, and 24 h shifts based on whether the shift began in the AM or PM. Differences in sleepiness were detected at the start and throughout the 12 h shifts when stratified by AM/PM status (all *p* values < 0.05). Regarding 24 h shifts, the only AM/PM differences in sleepiness detected occurred at 20 h into the shift (*p* = 0.0008). No AM/PM differences in sleepiness were detected for 8 h and 10 h shifts (all *p* values > 0.05). Similar to the findings for fatigue, among participants who reported the number of patient encounters during 24 h shifts, an increasing number of patient encounters was associated with greater sleepiness at all assessment time points during the shift (*p* < 0.0001; Supplemental Figure S11). On-shift sleep was associated with lower sleepiness at shift end for 12 h and 24 h shifts (*p* < 0.0001; Supplemental Table S1). Similar to fatigue, the effect of on-shift sleep on participant sleepiness at the end of shift was larger during 24 h vs. 12 h shifts, and larger for 24 h shifts that started in the AM vs. PM (*p* < 0.0001).

### 3.7. Difficulty with Concentration During Shifts

Participants reported low levels of difficulty with concentration at the start of 8 h, 10 h, 12 h, and 24 h shifts (mean 1.1; SD1.3; Supplemental Figure S7). No between-shift differences were detected (all *p* values > 0.05). Participants reported increased difficulty with concentration with increasing hours on shift for most shift durations, except for 24 h shifts, which again showed a sharp increase in difficulty with concentration at 16 h into the shift, followed by a sharp decrease at shift end (Supplemental Figure S7). The level of difficulty with concentration reported at shift end was higher than shift start for 10 h, 12 h, and 24 h shift durations (all *p* values < 0.05). The increase (delta) from shift start to shift end differed for 8 h shifts compared to 12 h shifts (*p* = 0.0189). No other shift durations had a difference in the increase (delta) from shift start to shift end (all *p* values > 0.05). Supplemental Figure S12 shows differences in difficulty with concentration during and at the end of 8 h, 10 h, 12 h, and 24 h shifts based on when the shift began, in the AM or PM. Participants reported higher levels of difficulty with concentration during and at the end of PM 12 h shifts versus AM 12 h shifts (all *p* values < 0.05); however, there were no AM/PM differences detected at shift start (*p* > 0.05). There were no AM/PM differences in difficulty with concentration detected for 8 h, 10 h, or 24 h shifts (all *p* values > 0.05). Like the findings reported for fatigue and sleepiness, among participants who reported the number of patient encounters during 24 h shifts, higher patient encounter volume was associated with greater difficulty with concentration throughout the shift (*p* < 0.0001; Supplemental Figure S13). On-shift sleep was associated with less difficulty with concentration at shift end for both 12 h and 24 h shifts (*p* < 0.05; Supplemental Table S1). Similar to fatigue and sleepiness outcomes, the effect of on-shift sleep during 24 h shifts was larger than that observed for 12 h shifts. In addition, the effect of on shift sleep lowering levels of difficulty with concentration was larger for 24 h shifts starting in the AM vs. PM (*p* < 0.0001).

## 4. Discussion

Findings from this secondary analysis provide insights regarding sleep and fatigue in relation to different duration shifts. The EMA-based data reveal patterns in fatigue, sleepiness, and difficulty with concentration not easily captured with cross-sectional methods. Because our data are linked to a large and diverse sample of EMS clinicians from across the U.S., generalizability is enhanced compared to our previous research that used similar EMA techniques with fewer EMS clinicians, fewer shifts, and less diversity in shift duration [9,10].

With frequent assessments during shifts, we showed that fatigue, sleepiness, and difficulty with concentration vary during shift work with the highest (poorest) reported levels associated with early morning hours (i.e., 03:00/04:00) reported during 24 h shifts. In addition, we showed that fatigue and sleepiness reported at the start and end of 12 h and 24 h shifts varied depending on whether a shift began in the antemeridian AM (00:00–11:59) or postmeridian PM (12:00–23:59) and whether participants napped during shift work. This finding suggests that EMS clinicians may be more at risk for fatigue or sleepiness related injury or error at the start of shifts that begin in the AM versus PM. Initially, this finding may appear counterintuitive, yet when considering circadian effects and sleep inertia on alertness and performance, with previous research showing 24 h rhythmicity in fatigue, sleepiness, mood, and cognitive performance with a nadir in the early morning hours, the findings are consistent with existing evidence [15,16,17]. Reporting a greater burden of fatigue and sleepiness in the early morning hours of 03:00 and 04:00 is not new; these data confirm what we already know about sleep pressure and fatigue and demonstrate the utility of a text message-based assessment during shift work conditions. In addition, our findings confirm what others have demonstrated previously, that sleep (napping) on duty can help mitigate feelings of fatigue and sleepiness [18]. Our findings add to our previous research [9,10] and provide new information on fatigue and related symptoms across different duration shifts, for which there is limited published information, especially in EMS and public safety occupations.

Sleep duration is one important component of sleep health [19]. The American Academy of Sleep Medicine, Sleep Research Society, and the National Sleep Foundation prescribe 7 to 9 h of sleep per night as adequate or ideal for adults, and sleep < 7 h as less optimal for health [20,21,22]. Approximately 25–30% of U.S. adults report inadequate sleep [22,23]. Shift workers like EMS and fire clinicians report inadequate and/or poor sleep at a higher prevalence than the general population [24,25]. We showed that while 51% of U.S. based EMS clinicians reported 6 or less hours of pre-shift sleep, many supplemented pre-shift sleep with on-shift sleep. We also showed that pre-shift sleep varies by shift duration and by the time of day when a shift begins (in the AM or PM). These data confirm that EMS clinician sleep is irregular and most fail to obtain nationally recommended sleep hours during a consolidated sleep opportunity. These findings are concerning, especially considering recent data that show the more irregular an individual’s sleep, the greater the risk of adverse cardiovascular health outcomes like myocardial infarction [26,27].

Fatigue and sleepiness associated with shift work cannot be fully eliminated; however, it can be managed [28]. Regular assessment of employee fatigue and indicators of sleep quality is one evidence-based component of fatigue risk management [1]. We used a novel, EMA-based approach to assess fatigue and the findings from this study shed light on specific time periods when fatigue and related symptoms like sleepiness are elevated and the evolution of fatigue and sleepiness within a given shift. Time of day affected fatigue, sleepiness, and difficulty with concentration at select time points during shifts. The highest documented levels of fatigue, sleepiness, and difficulty with concentration were identified during the early morning hours (i.e., at 03:00 and 04:00) during long duration shifts such as 24 h shifts. As mentioned previously, these findings align with known circadian variations in sleepiness and performance documented in other studies [29,30,31]. These data also reveal targets for intervention.

Long duration shifts beyond 24 h are becoming more prevalent in public safety operations across the U.S. [32,33,34,35]. Many U.S. based EMS and fire operations have adopted or added 48 h shifts. We do not have a definitive explanation for this trend or this specific duration (48 h). Potential reasons include difficulties with staffing, operational budget needs, and employee preference [32,33,34,35]. One systematic review of different shift durations showed that few studies have assessed fatigue or indicators of sleep immediately before, during, or after 48 h shifts or shifts longer than 24 h in duration [25]. Our understanding of the variations in fatigue and fatigue-related symptoms during 48 h or longer shifts is limited. In this study, we captured fatigue assessment data from 100 shifts equal to and greater than 36 h in duration, of which most were 48 h shifts. Given that the total number of these longer duration shifts is small relative to the more common 12 h, 24 h, and the shorter duration shifts documented in this study, we recommend caution in generalizing the findings from these shifts to all who use similar duration shifts. While limited, the data suggest that like the other shifts studied, fatigue and fatigue-related symptoms are clearly not stable during shift work. These symptoms vary considerably during work hours. For 48 h shifts, the patterns revealed for fatigue, sleepiness, and difficulty with concentration are like those seen with 24 h shifts with the potential for slightly higher levels on the second day of duty versus the first day. Additional research focused exclusively on shifts longer than 24 h in duration is needed with larger samples to draw definitive conclusions.

### Limitations

Our study faces several limitations. First, while our study sample is nationally representative, our data may not fully represent the experiences of all EMS/fire operations [36,37]. The specific roles, tasks, and patient care related workload of participants likely varies and may have an impact on fatigue and sleep outcomes measured in this study. This is especially true of individuals who work at fire-based operations where patient care related duties may represent a fraction of work during a typical shift. Findings from future studies may differ from what we report in this analysis based on the type and amount of work during shifts. Second, our protocol for data collection used a one week “on” and two weeks “off” rotation, which means our data do not represent all shifts worked over the study time period. In addition, participants may have intentionally or unintentionally not recorded a shift worked during the “on” data collection interval. Our findings therefore do not represent the full shift work-related experiences of participants. Third, a significant proportion of the data for this study were collected during the COVID-19 pandemic. Our findings may have been impacted. While the COVID-19 pandemic impacted sleep patterns of many adults [38,39], essential workers like EMS and fire clinicians typically maintained work schedules and recent data show that most who fit the employment and demographic characteristics of EMS/fire clinicians did not experience significant changes in sleep for extended periods of time [39]. The similarities between our findings (e.g., pre-shift sleep hours) and those of our prior studies (pre-pandemic) support this supposition [9,10].

## 5. Conclusions

Our findings show variability in shift scheduling, pre-shift and on-shift sleep, and wide variability in fatigue and fatigue-related symptoms during shifts of different durations, which is affected by the time of day when a shift begins and ends. Sub-group analyses of long duration 24 h shifts confirmed that greater patient volume (workload) is associated with less rest and sleep during shift work, greater fatigue, greater sleepiness, and greater difficulty with concentration. On-shift sleep (napping) during 12 h and 24 h shifts mitigates fatigue, sleepiness, and difficulty with concentration, with naps taken during 24 h shifts having greater impact. The real-time assessments of sleep hours and fatigue in this study reveal deficits in sleep health for EMS and fire personnel and critical time points for intervention during shifts when the risk to safety is high.

## Figures and Tables

**Table 1:** Shifts analyzed for primary outcomes.

Shift Duration	Number (%) of Shifts Analyzed with Text Message Data N = 4573	Percentage of Shifts with AM Start Times (Between 00:00–11:59)	Number of Individuals Documenting Shifts N = 388	Number of Agencies (Clusters) Contributing to Shifts Analyzed N = 35
8 h	252 (5.5%)	81.0%	59	28
10 h	239 (5.2%)	35.6%	39	19
12 h	1253 (27.4%)	67.8%	149	33
16 h	72 (1.6%)	65.3%	35	22
24 h	2657 (58.1%)	87.2%	286	29
36 h	26 (0.6%)	76.9%	23	17
48 h	67 (1.5%)	86.6%	31	14
60 h	3 (0.07%)	66.7%	3	2
72 h	4 (0.09%)	75.0%	3	2

h = hour; AM = antemeridian (00:00–11:59 h).

## Data Availability

Data for this research study reside with the U.S. Department of Transportation, National Highway Traffic Safety Administration (NHTSA) and all requests for data sharing/access to data should be directed to their office under contract number DTNH2215R00029.

## Supplementary Materials

The following supporting information can be downloaded at: https://www.mdpi.com/article/10.3390/ijerph22040573/s1, Supplemental File S1: Text message prompts/queries; Figure S2: Pre-shift sleep hours by shift duration; Figure S3: Pre-shift sleep hours by shift duration and by AM/PM; Figure S4: On-shift sleep by shift duration; Figure S5: On-shift sleep hours by shift duration and by AM/PM; Figure S6: Hours of sleep during shifts stratified by patient encounters during shift work (patient volume); Figure S7: EMS clinician fatigue, sleepiness, and difficulty with concentration at the start, during, and end of shifts stratified by shift duration; Figure S8: Differences in fatigue at start, during, and end of shift work by shift duration and AM/PM start times; Figure S9: Variation in fatigue at start, during, and end of 24-h shifts by quartiles of the number of patient encounters (patient volume) during shift work; Figure S10: Differences in sleepiness at start, during, and end of shift work by shift duration and AM/PM start times; Figure S11: Variation in sleepiness at start, during, and end of 24-h shifts by quartiles of the number of patient encounters (patient volume) during shift work; Figure S12: Differences in difficulty with concentration at start, during, and end of shift work by shift duration and AM/PM start times; Figure S13: Variation in difficulty with concentration at start, during, and end of 24-h shifts by quartiles of the number of patient encounters (patient volume) during shift work; Table S1: Association between on-shift sleep and fatigue, sleepiness, and difficulty with concentration reported at the end of shifts for 12-h and 24-h shifts.

## Abbreviations

The following abbreviations are used in this manuscript:

EMS	Emergency Medical Services
U.S.	United States
EMA	Ecological Momentary Assessment
PSQI	Pittsburgh Sleep Quality Index
AM	Ante Meridiem
PM	Post Meridiem
NC	North Carolina
SD	Standard Deviation
