# Supplementary material for: Variations in Sleep, Fatigue, and Difficulty with Concentration Among Emergency Medical Services Clinicians During Shifts of Different Durations
Source: Int J Environ Res Public Health. 2025 Apr 6;22(4):573. doi: 10.3390/ijerph22040573 (PMC12026690; doi:10.3390/ijerph22040573)
Supplement: Supplementary file 1 [file ijerph-22-00573-s001.zip › ijerph-3500574-supplementary.pdf]

Supplemental File

Text message prompts / queries

Start of shift text queries (typically 15 minutes after shift began)

How many hours did you sleep since Thursday, December 28, 12:00 Eastern Time (specify in half hours, ex, 2.5. No sleep-0)?

What time did you lay down to sleep? Answer in military hours (e.g. 1530)

What time did you get up? Answer in military hours (e.g. 0530)

How many times was your sleep disrupted? (Answer numerically, 0=none)

Rate SLEEPINESS now (0-5) with 0=Not At All and 5=Very Much. Text “slp” for definition of sleepiness

Rate Fatigue now (0-5) with 0=Not At All and 5=Very Much. Text “ftg” for definition of fatigue

How difficult is it to CONCENTRATE now (0-5) with 0=Not At All and 5=Very Much. Text “ccgt” for definition of concentrate

During shift text queries (every 4 hours)

How many hours did you sleep since Thursday, December 28, 12:00 Eastern Time (specify in half hours, ex, 2.5. No sleep-0)?

What time did you lay down to sleep? Answer in military hours (e.g. 1530)

What time did you get up? Answer in military hours (e.g. 0530)

How many times was your sleep disrupted? (Answer numerically, 0=none)

Rate SLEEPINESS now (0-5) with 0=Not At All and 5=Very Much. Text “slp” for definition of sleepiness

Rate FATIGUE now (0-5) with 0=Not At All and 5=Very Much. Text “ftg” for definition of fatigue

How difficult is it to CONCENTRATE now (0-5) with 0=Not At All and 5=Very Much. Text “ccgt” for definition of concentrate

End of shift text queries

How many hours did you sleep DURING your shift? (specify in half hours, ex, 2.5. No sleep-0)?

Rate FATIGUE now (0-5) with 0=Not At All and 5=Very Much. Text “ftg” for definition of fatigue

Rate SLEEPINESS now (0-5) with 0=Not At All and 5=Very Much. Text “slp” for definition of sleepiness

How difficult is it to CONCENTRATE now (0-5) with 0=Not At All and 5=Very Much. Text “ccgt” for definition of concentrate

Estimate the total number of patients you saw during your shift

Were you injured during your shift (Yes or No)

If participants reported injury

Please describe your TYPE of injury and body part affected. Text "inj" for definition of injury.

Rate Fatigue AT TIME OF INJURY (0-5) with 0=Not At All and 5=Very Much

Rate Sleepiness AT TIME OF INJURY (0-5) with 0=Not At All and 5=Very Much

How difficult was it to CONCENTRATE AT TIME OF INJURY with 0=Not At All and 5=Very Much

Be sure to follow your employer's protocol for injury reporting. These text messages are for research purposes only.

Thanks for completing the End of Shift questions. You have no future shifts in the schedule. Visit www.XXXXXXXXXXXXX to enter shifts.

Sources for text message / query structure / content:

Patterson PD, Moore CG, Guyette FX, Doman JM, Sequeira D, Werman HA, Swanson D, Hostler D, Lynch J, Russo L, Hines L, Swecker K, Runyon MS, Buysse DJ. Fatigue mitigation with SleepTrackTXT2 in air medical emergency care systems: study protocol for a randomized controlled trial. Trials. 2017 Jun 5;18(1):254. doi: 10.1186/s13063-017-1999-z. PubMed PMID: 28583143; PubMed Central PMCID: PMC5460424.

Patterson PD, Buysse DJ, Weaver MD, Doman JM, Moore CG, Suffoletto BP, McManigle KL, Callaway CW, Yealy DM. Real-time fatigue reduction in emergency care clinicians: The SleepTrackTXT randomized trial. Am J Ind Med. 2015 Oct;58(10):1098-113. doi: 10.1002/ajim.22503. Epub 2015 Aug 25. PubMed PMID: 26305869; PubMed Central PMCID: PMC4573891.

Patterson PD, Moore CG, Weaver MD, Buysse DJ, Suffoletto BP, Callaway CW, Yealy DM. Mobile phone text messaging intervention to improve alertness and reduce sleepiness and fatigue during shiftwork among emergency medicine clinicians: study protocol for the SleepTrackTXT pilot randomized controlled trial. Trials. 2014 Jun 21;15:244. doi: 10.1186/1745-6215-15-244. PubMed PMID: 24952387; PubMed Central PMCID: PMC4080698.

Figure S2: Pre-shift sleep hours by shift duration

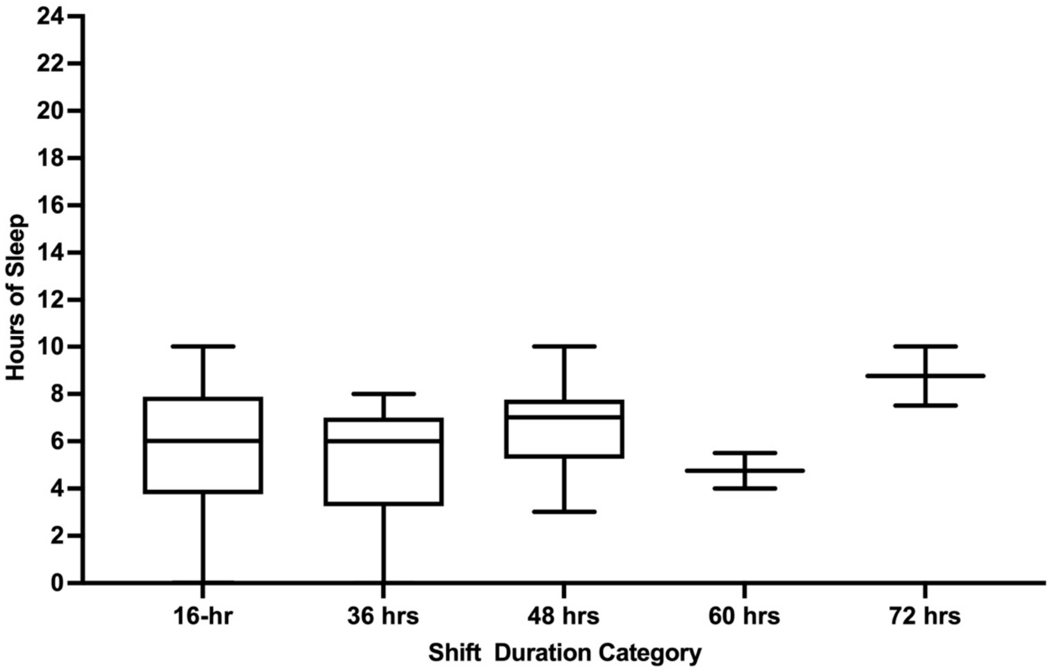

Figure S2 footnotes: 16-hr shift (14 to 19.99 hours), 36-hr shift (30 to 40.99 hours), 48-hr shift (41 to 54.99 hours), 60-hr shift (55 to 65.99 hours), and 72-hr shift (≥66 hours). Box = median and interquartile range. Whiskers = minimum and maximum.

Figure S3: Pre-shift sleep hours by shift duration and by AM/PM

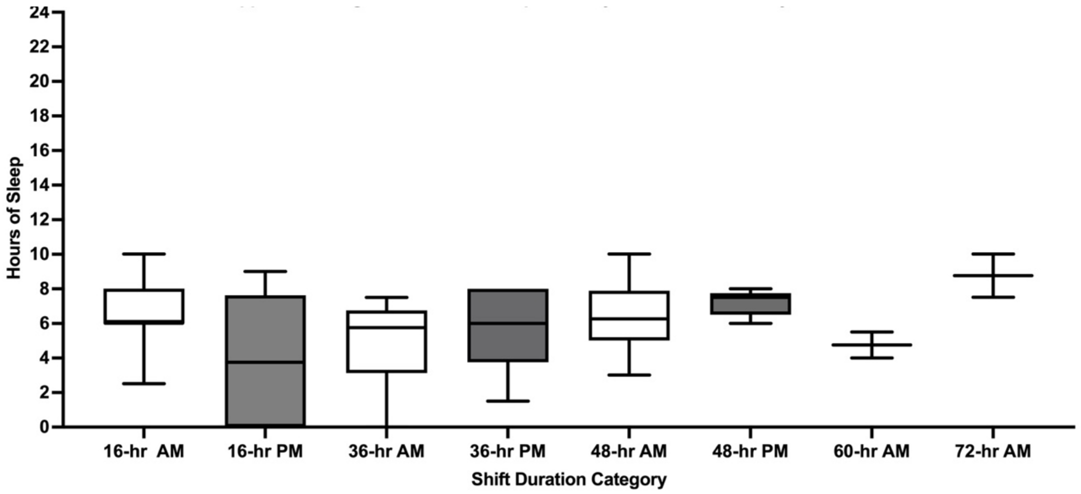

Figure S3 footnotes: 16-hr shift (14 to 19.99 hours), 36-hr shift (30 to 40.99 hours), 48-hr shift (41 to 54.99 hours), 60-hr shift (55 to 65.99 hours), and 72-hr shift (≥66 hours). AM=antemeridian (00:00-11:59). PM=postmeridian (12:00-23:59). Box = median and interquartile range. Whiskers = minimum and maximum.

Figure S4: On-shift sleep by shift duration

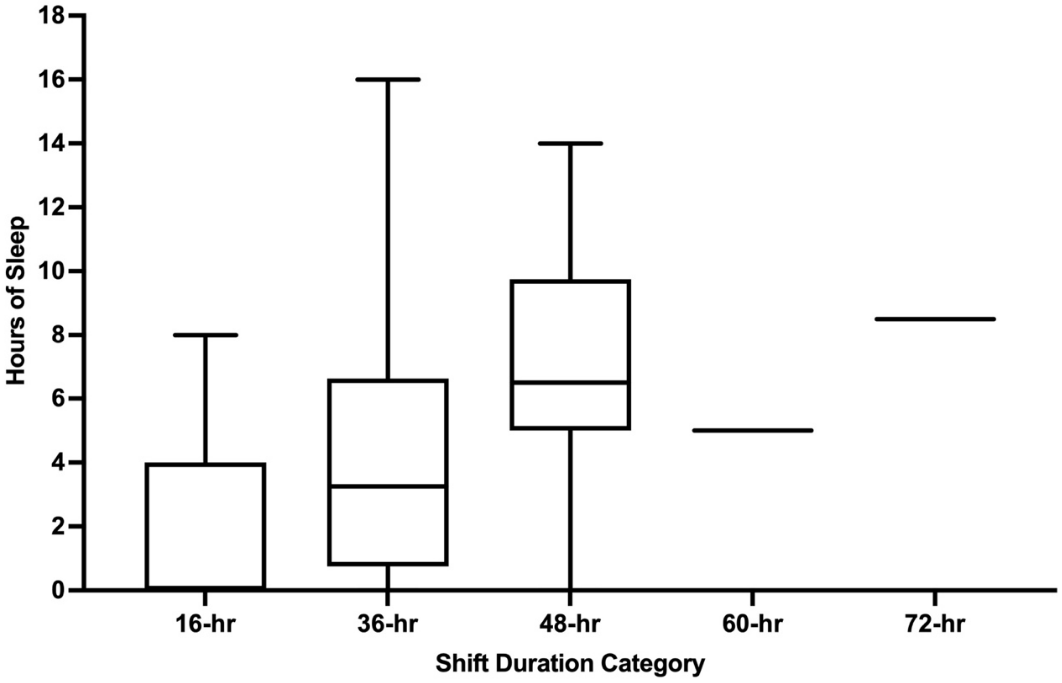

Figure S4 footnotes: 16-hr shift (14 to 19.99 hours), 36-hr shift (30 to 40.99 hours), 48-hr shift (41 to 54.99 hours), 60-hr shift (55 to 65.99 hours), and 72-hr shift (≥66 hours). Box = median and interquartile range. Whiskers = minimum and maximum.

Figure S5: On-shift sleep hours by shift duration and by AM/PM

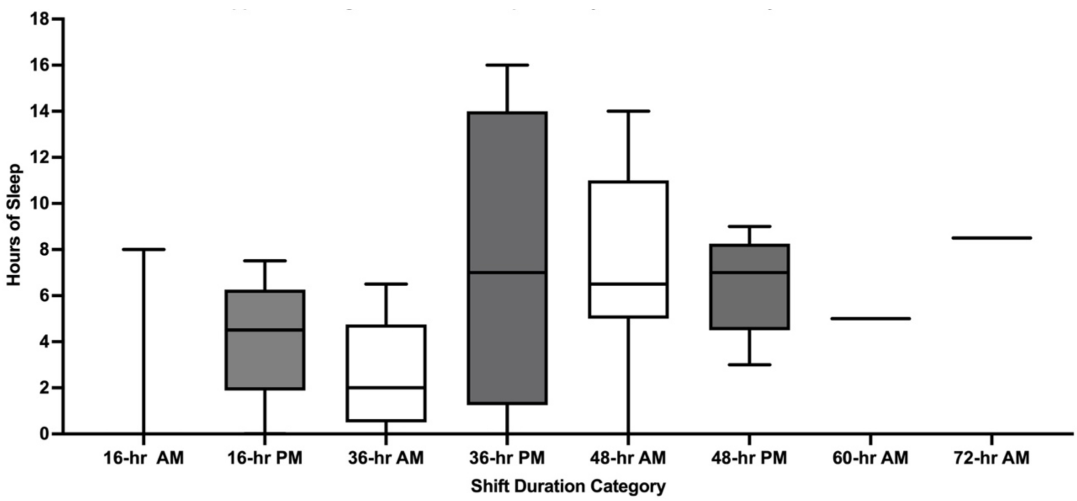

Figure S5 footnotes: 16-hr shift (14 to 19.99 hours), 36-hr shift (30 to 40.99 hours), 48-hr shift (41 to 54.99 hours), 60-hr shift (55 to 65.99 hours), and 72-hr shift (≥66 hours). AM=antemeridian (00:00-11:59). PM=postmeridian (12:00-23:59). Box = median and interquartile range. Whiskers = minimum and maximum.

Figure S6: Hours of sleep during shifts stratified by patient encounters during shift work (patient volume)

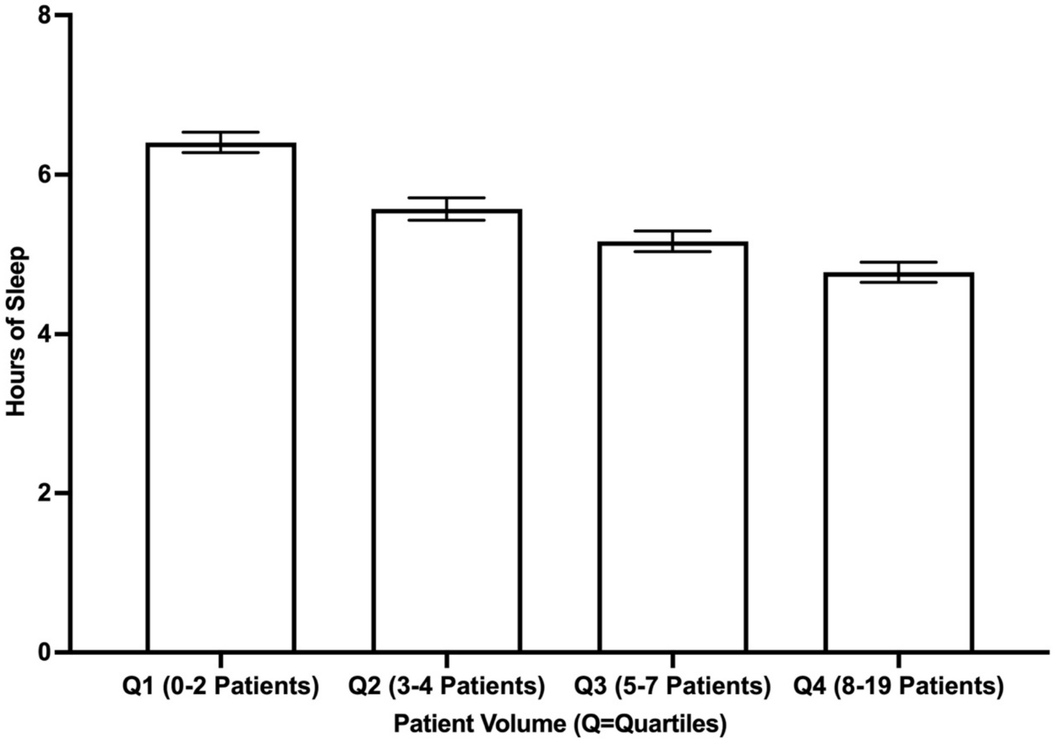

Figure S6 footnotes: Bars equal mean with whiskers as standard error.

Figure S7: EMS clinician fatigue, sleepiness, and difficulty with concentration at the start, during, and end of shifts stratified by shift duration

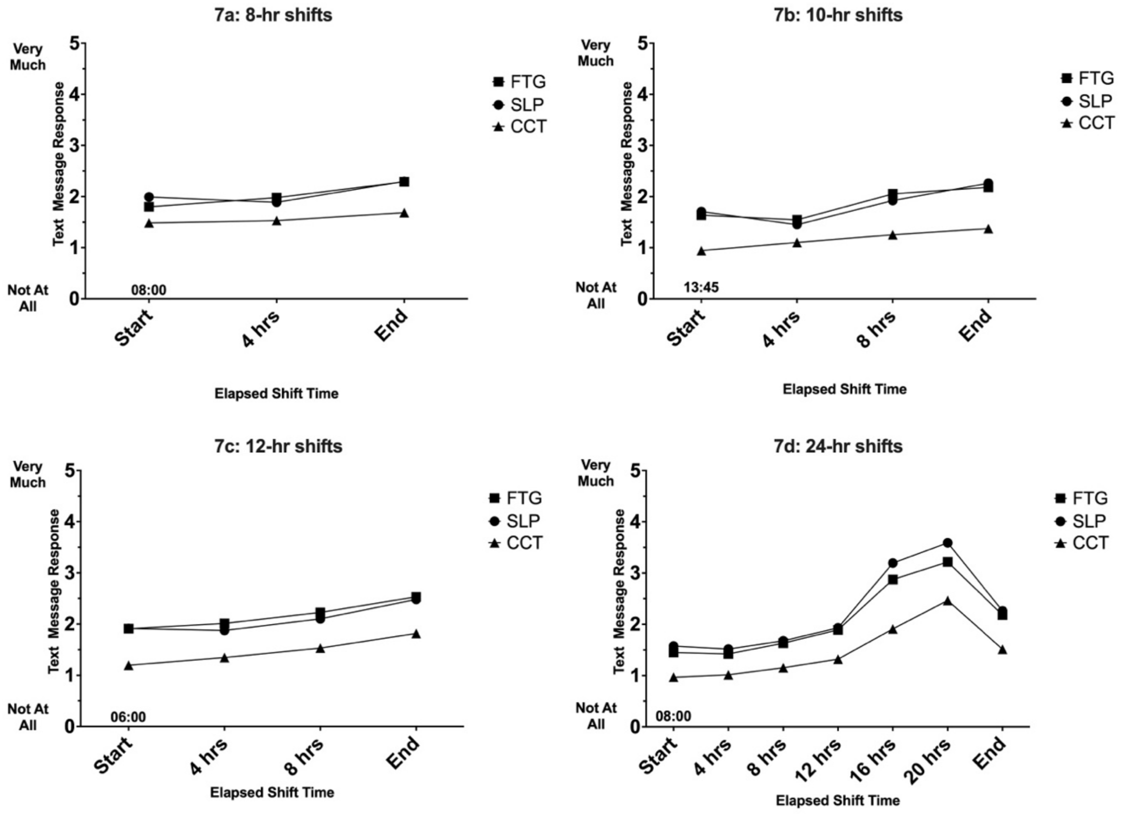

Figure S7 footnotes: Interpretation of Y-axis scaling: 0= "Not at all" and 5= "Very much." Figure A7a. 8-hr shifts were inclusive of any shift of <9.5 hours in length. The most frequent start time for an 8-hr shift was at 08:00 (27%). Figure A7b. 10-hr shifts were inclusive of any shift of 9.5 to <11 hours in length. The most frequent start time for a 10-hr shift was at 13:25 (18%). Figure A7c. 12-hr shifts were inclusive of any shift of 11 to <14 hours in length. The most frequent start time for a 12-hr shift was at 06:00 (19%). Figure A7d. 24-hr shifts were inclusive of any shift of 20 to <30 hours in length. The most frequent start time for a 24-hr shift was at 08:00 (34%)

Figure S8: Differences in fatigue at start, during, and end of shift work by shift duration and AM/PM start times

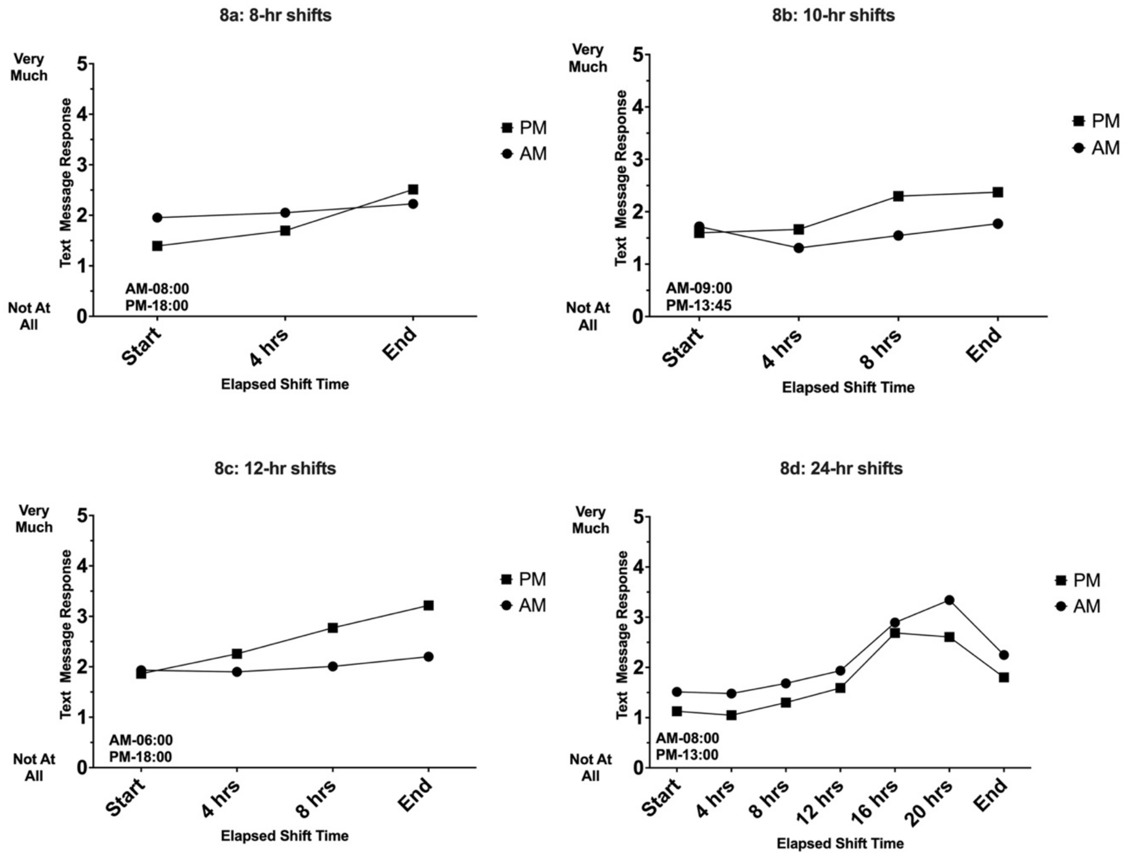

Figure S8 footnotes: Interpretation of Y-axis scaling: 0= "Not at all" and 5= "Very much." AM=antemeridian (00:00-11:59). PM=postmeridian (12:00-23:59). Figure A8a. 8-hr shifts were inclusive of any shift of <9.5 hours in length. The most frequent start time of AM starting 8-hr shifts was at 08:00 (34%). The most frequent start time of PM starting 8-hr shifts was at 18:00 (35%). Figure A8b. 10-hr shifts were inclusive of any shift of 9.5 to <11 hours in length. The most frequent start time of AM starting 10-hr shifts was at 09:00 (19%). The most frequent start time of PM starting 10-hr shifts was at 13:45 (27%). Figure A8c. 12-hr shifts were inclusive of any shift of 11 to <14 hours in length. The most frequent start time of AM starting 12-hr shifts was at 06:00 (29%). The most frequent start time of PM starting 12-hr shifts was at 18:00 (40%). Figure A8d. 24-hr shifts were inclusive of any shift of 20 to <30 hours in length. The most frequent start time of AM starting 24-hr shifts was at 08:00 (39%). The most frequent start time of PM starting 24-hr shifts was at 13:00 (96%).

Figure S9: Variation in fatigue at start, during, and end of 24-hr shifts by quartiles of the number of patient encounters (patient volume) during shift work

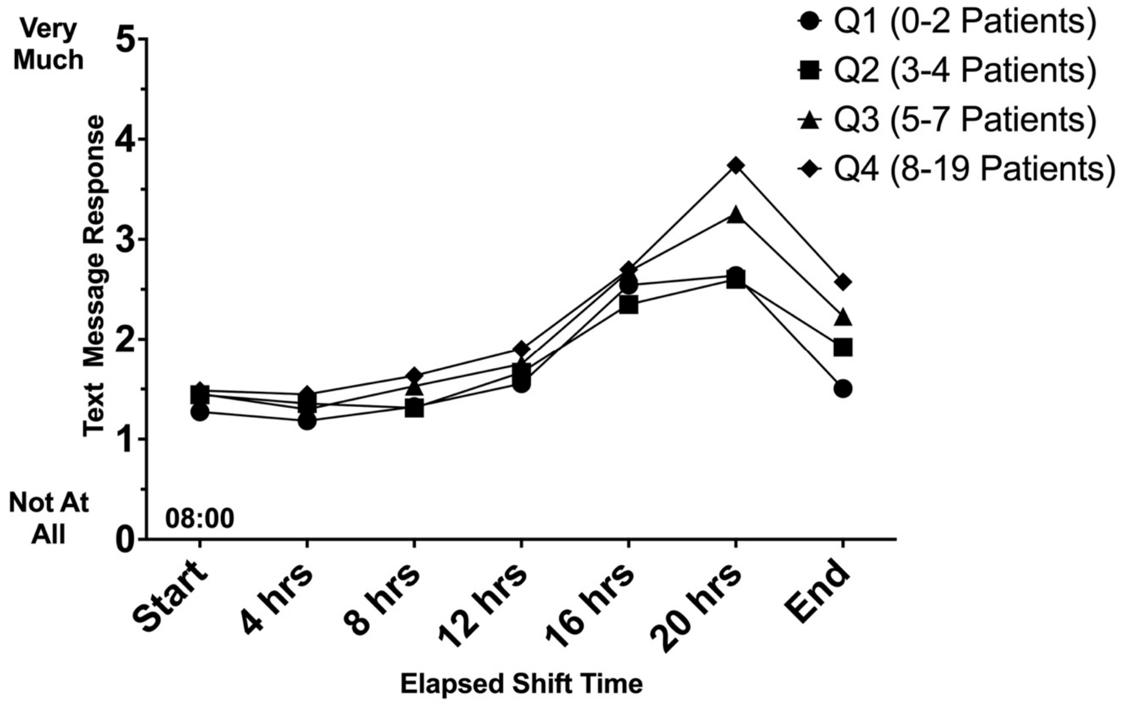

Figure S9 footnotes: Interpretation of Y-axis scaling: 0= “Not at all” and 5= “Very much.” The most frequent start time for a 24-hr shift was at 08:00 (34%).

Figure S10: Differences in sleepiness at start, during, and end of shift work by shift duration and AM/PM start times

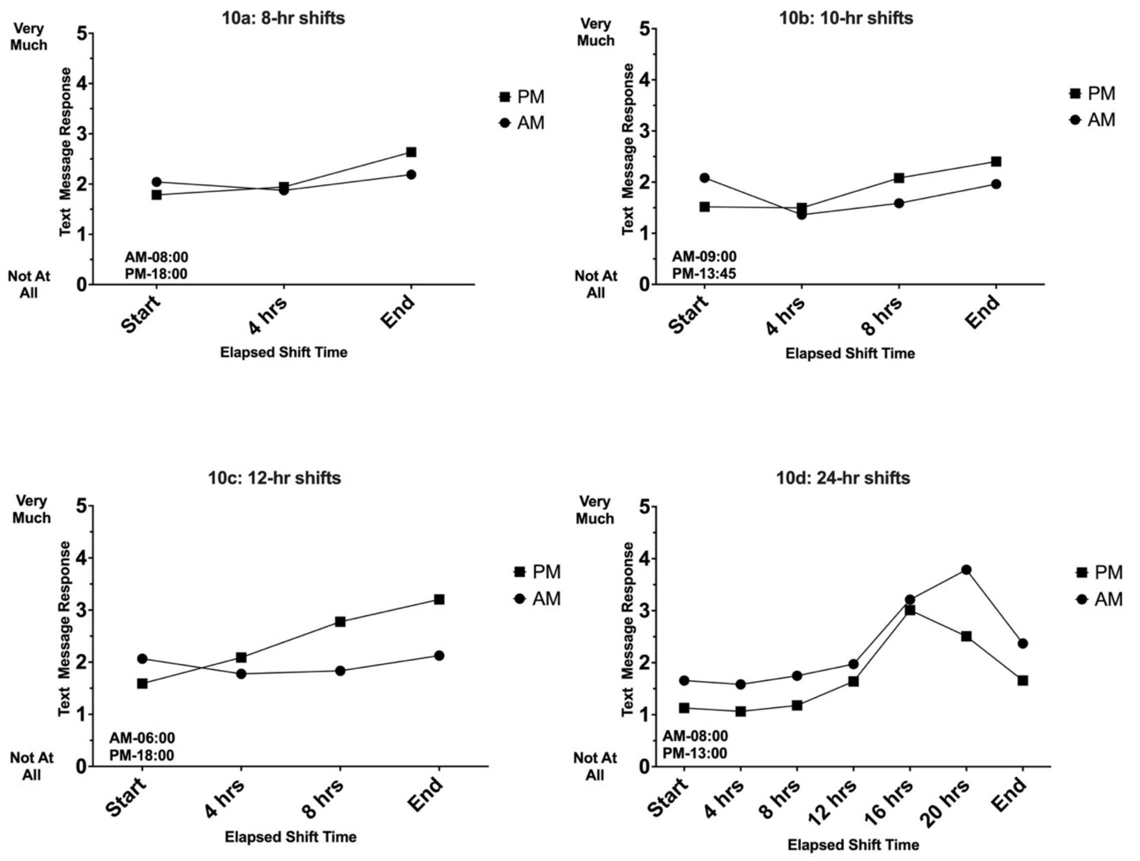

Figure S10 footnotes: Interpretation of Y-axis scaling: 0= "Not at all" and 5= "Very much." AM=antemeridian (00:00-11:59). PM=postmeridian (12:00-23:59). Figure A10a. 8-hr shifts were inclusive of any shift of <9.5 hours in length. The most frequent start time of AM starting 8-hr shifts was at 08:00 (34%). The most frequent start time of PM starting 8-hr shifts was at 18:00 (35%). Figure A10b. 10-hr shifts were inclusive of any shift of 9.5 to <11 hours in length. The most frequent start time of AM starting 10-hr shifts was at 09:00 (19%). The most frequent start time of PM starting 10-hr shifts was at 13:45 (27%). Figure A10c. 12-hr shifts were inclusive of any shift of 11 to <14 hours in length. The most frequent start time of AM starting 12-hr shifts was at 06:00 (29%). The most frequent start time of PM starting 12-hr shifts was at 18:00 (40%). Figure A10d. 24-hr shifts were inclusive of any shift of 20 to <30 hours in length. The most frequent start time of AM starting 24-hr shifts was at 08:00 (39%). The most frequent start time of PM starting 24-hr shifts was at 13:00 (96%).

Figure S11: Variation in sleepiness at start, during, and end of 24-hr shifts by quartiles of the number of patient encounters (patient volume) during shift work

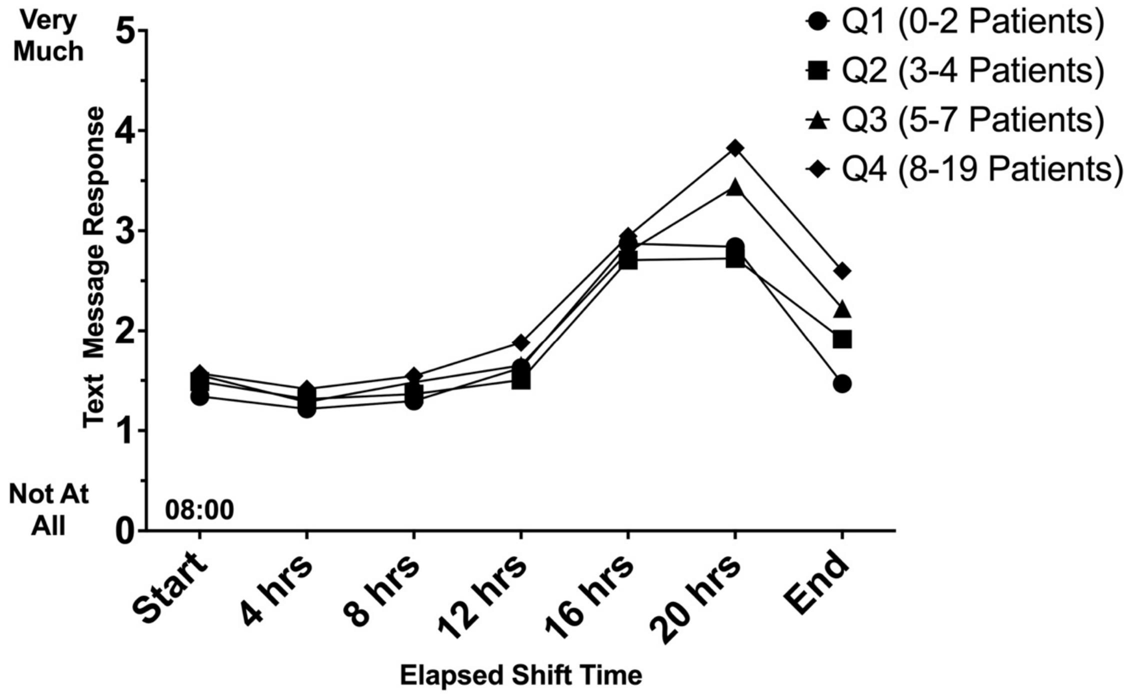

Figure S11 footnotes: Interpretation of Y-axis scaling: 0= “Not at all” and 5= “Very much.” The most frequent start time for a 24-hr shift was at 08:00 (34%).

Figure S12: Differences in difficulty with concentration at start, during, and end of shift work by shift duration and AM/PM start times

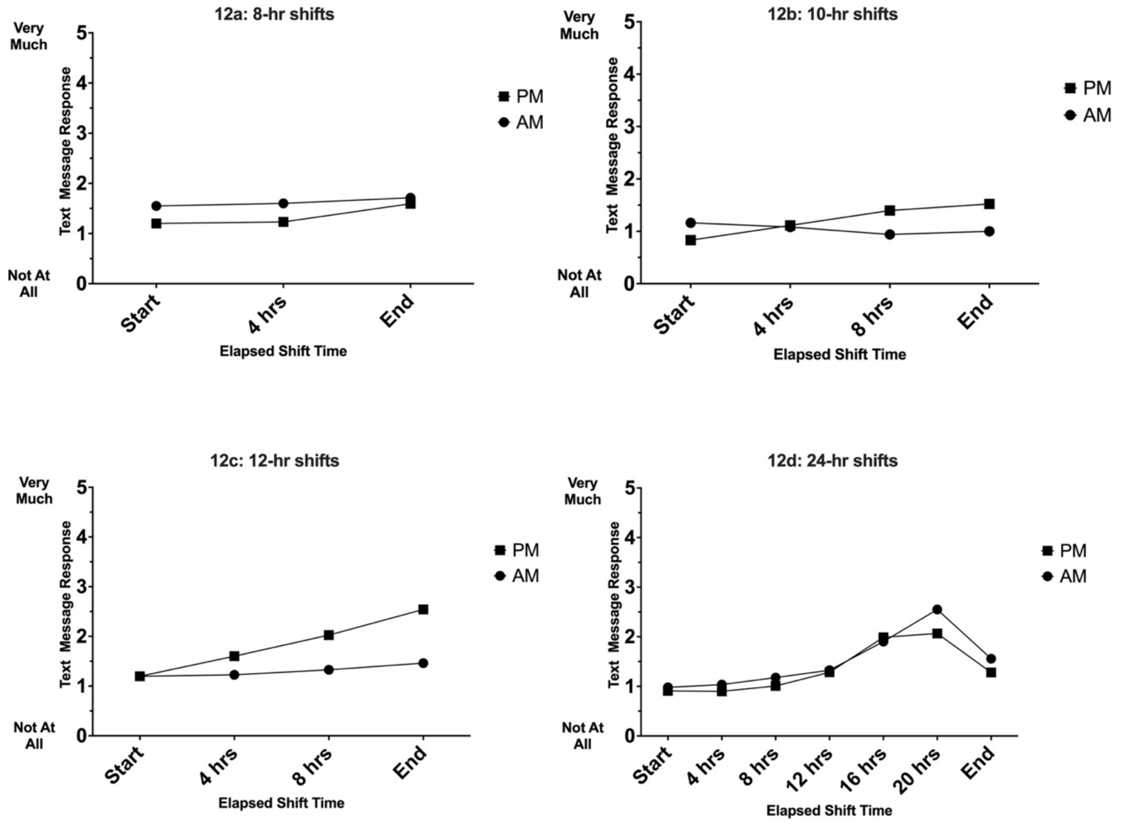

Figure S12 footnotes: Interpretation of Y-axis scaling: 0= "Not at all" and 5= "Very much." AM=antemeridian (00:00-11:59). PM=postmeridian (12:00-23:59). Figure A12a. 8-hr shifts were inclusive of any shift of <9.5 hours in length. The most frequent start time of AM starting 8-hr shifts was at 08:00 (34%). The most frequent start time of PM starting 8-hr shifts was at 18:00 (35%). Figure A12b. 10-hr shifts were inclusive of any shift of 9.5 to <11 hours in length. The most frequent start time of AM starting 10-hr shifts was at 09:00 (19%). The most frequent start time of PM starting 10-hr shifts was at 13:45 (27%). Figure A12c. 12-hr shifts were inclusive of any shift of 11 to <14 hours in length. The most frequent start time of AM starting 12-hr shifts was at 06:00 (29%). The most frequent start time of PM starting 12-hr shifts was at 18:00 (40%). Figure A12d. 24-hr shifts were inclusive of any shift of 20 to <30 hours in length. The most frequent start time of AM starting 24-hr shifts was at 08:00 (39%). The most frequent start time of PM starting 24-hr shifts was at 13:00 (96%).

Figure S13: Variation in difficulty with concentration at start, during, and end of 24-hr shifts by quartiles of the number of patient encounters (patient volume) during shift work

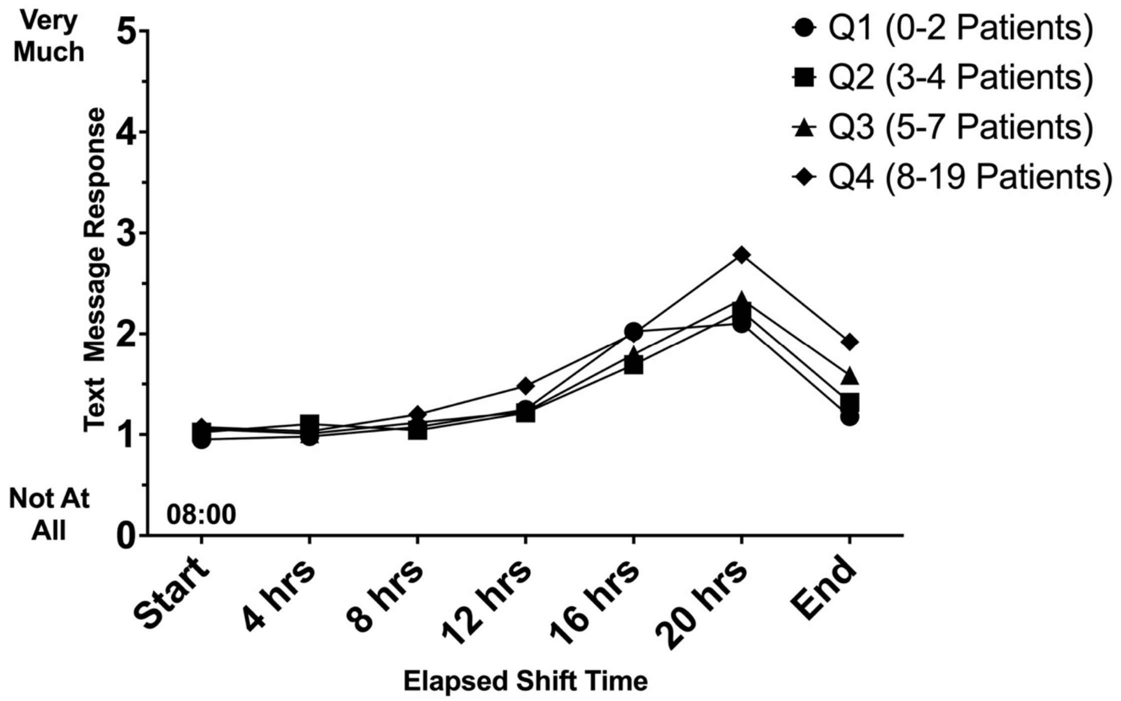

Figure S13 footnotes: Interpretation of Y-axis scaling: 0= “Not at all” and 5= “Very much.” The most frequent start time for a 24-hr shift was at 08:00 (34%).

Table S1. Association between on-shift sleep and fatigue, sleepiness, and difficulty with concentration reported at the end of shifts for 12-hr and 24-hr shifts

	**Model 1**<br>**12-hr shifts**<br><br>**Outcome:**<br>**End of shift CCT**<br><br>n=757	**Model 2**<br>**12-hr shifts**<br><br>**Outcome:**<br>**End of shift FTG**<br><br>n=836	**Model 3**<br>**12-hr shifts**<br><br>**Outcome:**<br>**End of shift SLP**<br><br>n=800	**Model 4**<br>**24-hr shifts**<br><br>**Outcome:**<br>**End of shift CCT**<br><br>n=1,457	**Model 5**<br>**24-hr shifts**<br><br>**Outcome:**<br>**End of shift FTG**<br><br>n=1,590	**Model 6**<br>**24-hr shifts**<br><br>**Outcome:**<br>**End of shift SLP**<br><br>n=1,525
Overall Model<br><br>Model predictor variables: on-shift sleep in hours	$\beta'_{\text{on-shift sleep}} = -0.07$, p=0.03	$\beta'_{\text{on-shift sleep}} = -0.12$, p<0.0001	$\beta'_{\text{on-shift sleep}} = -0.15$, p<0.0001	AM shifts<br>$\beta'_{\text{on-shift sleep}} = -0.35$*<br>p<0.0001<br><br>PM shifts<br>$\beta'_{\text{on-shift sleep}} = -0.24$*<br>p<0.0001	$\beta'_{\text{on-shift sleep}} = -0.43$, p<0.0001	AM shifts<br>$\beta'_{\text{on-shift sleep}} = -0.44$*<br>p<0.0001<br><br>PM shifts<br>$\beta'_{\text{on-shift sleep}} = -0.34$*<br>p<0.0001

Table S1 footnotes: β' = the estimate of the slope for on-shift sleep from linear mixed models. CCT = difficulty with concentration. FTG = fatigue. SLP = sleepiness. AM=antemeridian (00:00-11:59). PM=postmeridian (12:00-23:59). *Coefficients presented here are from a linear mixed model that included an interaction term for AM/PM status with number of hours of sleep achieved on shift where the interaction was significant at p<0.05 (overall model: Model4 and Model6). If the interaction term was not significant, the coefficient is from the model without the interaction term included. All models included a random effect for Agency and accounted for clustering of participants within each Agency. The outcome variables were CCT, FTG, and SLP at the end of shift treated as continuous variables, respectively.
